# Incorporating new technologies in breeding plans for South African goats in harsh environments

**DOI:** 10.1093/af/vfad040

**Published:** 2023-10-13

**Authors:** Carina Visser, Margaretha A Snyman

**Affiliations:** Department of Animal Science, University of Pretoria, P/Bag X28, Pretoria, 0028, South Africa; Grootfontein Agricultural Development Institute, P/Bag X529, Middelburg, EC, 5900, South Africa

**Keywords:** adaptation, genomics, indigenous, selection

**Implications**

- Angora and meat producing goats play an important role in food security and sustainable livelihoods of livestock producers in South Africa. To maintain their role, it is important to select animals that can thrive in the harsh South African climate, especially under the envisioned climate change conditions.
- Several phenotypes have been identified as selection criteria for adaptation. These include litter size at birth, litter size at weaning and litter weight at weaning (reproduction), weaning weight (growth), fecal egg counts, FAMACHA score, packed cell volume, tick counts, hair length, and coat type (endo- and ecto-parasite resistance) and coat type, coat thickness, color of skin and coat, rectal temperature (heat tolerance). Many of these phenotypic indicator traits have limitations such as low heritabilities or being difficult to measure.
- The application of new technologies in breeding and selection is under-utilized in goats in general, and especially in South African goats. The use of validated causative mutations affecting traits relating to adaptation to harsh environments in selection programs would facilitate genetic progress in herds where phenotypic recording is challenging.
- In addition, increased investments by various role players in applying new technology in the systematic collection of phenotypic indicator traits (especially for traits related to adaptation), can be used to improve the rate of animal improvement in the South African goat breeds.

## Introduction

Goats play an important role in the agricultural sector in South Africa and are raised under varying climatic conditions in most parts of the country, almost exclusively under extensive farming systems. The animals are subjected to a range of adverse conditions ranging from hot, dry summers, limited and seasonal food sources, external parasites and internal parasite challenges during the rainy seasons, to very cold winters.

The most populous goat breed in South Africa is the mohair-producing Angora goat, followed by the commercially farmed, meat producing Boer goat, Kalahari Red goat, and Savannah goat breeds. These commercial breeds constitute between 30% and 40% of the South African goat population, with the exotic dairy goats (Saanen and Toggenburg) making up less than 1% of South Africa’s goat numbers. The origin and description of these commercial breeds, as well as their production and reproductive performance standards, distribution over the country and breed numbers (where available) were summarized by Snyman (2014).

Almost 70% of the meat goats in South Africa are, however, indigenous goats. Even though their role and relative importance are dependent on many variables (e.g., region, cultural values, and socio-economic status), indigenous goats generally contribute to meat, milk, fiber, and hide production in rural communities. Four indigenous goat ecotypes are recognized in South Africa, namely the Nguni type, Eastern Cape Xhosa type, Northern Cape, lob eared, speckled type and the Kunene type (Snyman, 2014). These ecotypes were registered collectively in 2006 during the establishment of the Indigenous Veld Goat Society, and breeders participate in official pedigree and performance recording. On the contrary, goats kept in traditionally managed rural or communal systems are often not formally classified within any of the ecotypes and are considered phenotypically nondescript.

For an animal to optimally produce and reproduce, it should be adapted to its production environment. The animal must be able to survive, thrive, produce and reproduce without excessively high input costs or managerial demands in terms of feed and health care (Burrow, 2015). The long-term solution to obtain adapted animals that can withstand endemic stressors, is to breed animals that are adapted to their specific environment. Considerable progress was made in goat breeding technologies from the 1960s with the first publication of heritability estimates, which was followed by the implementation of estimated breeding values (Salgado Pardo, 2022). During the past decade, the goat reference genome was developed, commercial SNP arrays became available and whole genome sequencing techniques were applied in goat research (Salgado Pardo, 2022).

Breeding for efficiency, especially in a climate-change environment, has become a new challenge for most livestock species. The feasibility of incorporating new technologies in breeding plans for South African goats in harsh environments is discussed here.

### Breeding objectives and selection criteria

Breeding for increased efficiency and adaptability under harsh environments involves a wide array of breeding objectives and traits, from routinely measured traits such as weights and reproduction to less-common traits such as heat resistance and faecal egg counts. The rate of genetic progress expected can differ vastly between these traits, and will be discussed in more detail for some of the traits.

### Reproductive traits

Reproductive ability in the presence of endemic stressors is one of the aspects that define an animal’s ability to adapt to harsh environments (Burrow, 2015). Reproduction is a complex trait and there are various measures and definitions for the components of reproduction, but the most regularly used measures are litter size at birth, litter size at weaning and litter weight at weaning, with the latter two traits including maternal ability. Litter weight at weaning as a lifetime trait of a doe has been indicated as a composite trait that is a good indicator of overall reproductive efficiency (Snyman, 2020). This trait includes both a maternal and direct component, as it incorporates both the number of kids, as well as the weaning weight of the kids produced. Although reproductive performance is recorded by breeders participating in the National Small Stock Improvement Scheme, in many instances only the litter size at weaning is recorded. Litter size at birth and does that did not produce a kid are not recorded, which lead to biased breeding values being estimated. In challenging environments, care must be taken that selection pressure on yield traits (e.g., body weight, fleece production) does not adversely impact on reproductive efficiency.

### Growth traits

The performance trait that is the most indicative of adaptation is growth rate, especially pre- and postweaning growth rate. Preweaning growth rate also depends on the milk production and maternal ability of the dam, while postweaning growth is mainly a function of the additive genetic component and it impacts largely on age at marketing, selection opportunities and future reproduction of the maiden doe. Weaning and postweaning weight are recorded by breeders participating in the National Small Stock Improvement Scheme.

### Disease resistance

Disease resistance is often used as a generic term that refers to resistance to infection, and thus to the animal’s ability to restrain a pathogen or parasite lifecycle (Bishop and Morris, 2007). It can be used to describe the animal’s response to both internal and external parasite infestation, as well as bacterial, viral, or metabolic diseases. It is commonly accepted that disease resistance has a genetic background with variation between hosts, and that this could be used to select and breed animals with superior resistance.

Gastrointestinal parasites have a direct adverse effect on animal reproduction efficiency, health and performance. The generally accepted phenotypic measures indicating relative nematode resistance include fecal egg counts (FEC), FAMACHA scores, dag scores and packed cell volume (PCV), depending upon the specific nematode involved. A summary of potential phenotypes as indicators of resistance to gastrointestinal nematodes, was provided by Mpofu et al. (2022). These include a range of measurements, such as parasitological (e.g., FEC, worm counts), immunological (e.g., levels of Immunoglobulin IgA, IgE, IgG, and IgM antibodies) and pathological (e.g., PCV) phenotypes.

Apart from ticks transmitting disease-causing pathogens to animals, which may cause serious losses in productivity and may lead to high mortality levels, their bites also cause damage to the skin and tissues (Cloete et al., 2021). The counting of ticks or lice on the body of an animal is an accurate indication of external parasite infestation. Coat traits, such as skin thickness, hair length, and coat type (smoothness of the coat) are significantly related to tick resistance and are also used as phenotypic indicators for increased resistance to ecto-parasites in cattle (Marufu et al., 2011), although no research in this regard was performed on goats (Burrow, 2015). These coat traits are not dependent on the presence and level of the stressor (as is tick counts), and it is thus easier to select for.

### Heat resistance

Heat stress results in a decrease in growth, milk, and meat production and immune response in goats. There exists significant variation regarding heat resistance both between breeds, but also within a breed, based on a combination of the individual’s inherent physiological, morphological, behavioral and genetic responses to heat stress. Phenotypic measurements to assess heat tolerance may include coat type, coat thickness, number and length of hairs, pigmentation (color) of the skin and coat, number of sweat glands, sweating rate, rectal temperature, skin temperature, heart rate, respiratory rate and various blood parameters (Castanheira et al. 2010). Rectal temperature has traditionally been regarded as a good indicator of an animal’s ability to withstand heat stress. This trait has favorable genetic correlations with improved weight gain, and reproductive efficiency. Some easier to measure traits, such as coat type (woolly vs. sleek) and coat color (light vs. dark) could result in improved heat tolerance, and would also have a favorable impact on lifetime female reproduction. The favorable correlations between growth traits and heat tolerance traits indicate that when the ability of an animal to handle heat stress improves, it also increases its genetic ability to grow (Burrow, 2015).

### Challenges to attain genetic progress

Burrow (2015) suggested that measuring phenotypes in commercial production environments is the single biggest limitation to the genetic improvement of adaptive traits in livestock. The nature of the trait (often discontinuous and poorly correlated with actual field resistance), and the influence of the environment, as well as the health status of the host on the level of the challenge, further complicates selection and breeding for adaptation. This is especially true for traits such as nematode resistance, where there are many phenotypic indicator traits that could be used, but no single trait is a true measure of host resistance. The molecular mechanism behind host resistance against nematodes is complex and involves various levels that still need to be precisely deciphered (Shrivastava et al., 2022). Therefore, a combination of phenotypic indicators such as fecal egg count, packed cell volume and immunoglobulin levels should yield a more accurate estimation of the genetic level of nematode resistance (Mpofu et al., 2022).

Tick and lice counts can be difficult to record under extensive farming conditions. Certain environmental factors also have an influence on the parasite load on animals. Season, rainfall and temperature directly influence parasitic load, while sex and age of animals might influence the susceptibility of cattle (Mapholi et al., 2014). Many possible indicator traits for heat stress are impractical for farmers to measure, i.e., heart and pulse rates, or hormone and electrolyte levels. Rectal temperature is marginally easier to record, but is still invasive and difficult in extensive systems. Coat type and color, however, are easy to record and have recorded heritability estimates ranging from 0.08 to 0.65 in cattle (Burrow, 2015), and thus fast genetic progress can be expected.

Even though the phenotypic indicator traits for reproduction, namely litter size at birth and litter size at weaning are direct measurements of the breeding objective, it is not always easy to accurately record under extensive farming conditions. Kids that are stillborn or kids that died soon after birth are not recorded in many instances. Recording of pedigrees under extensive farming conditions are also difficult and prone to mis-allocation of offspring to dams. Furthermore, reproductive traits have lower heritability estimates and are expressed at a later stage in life.

Noncomprehensive summaries of heritability estimates for production, reproduction, and disease resistance traits related to adaptation in Angora and Boer goats are given in Tables 1 and 2, respectively. Similar heritabilities for the other South African goat breeds, as well as parameters for tick and heat resistance traits for the South African breeds, are not available in literature. Heritabilities ranging from 0.06 to 0.62 are reported for Merino sheep at weaning and hogget age for rectal temperature recorded at various times of the day (Rose and Pepper, 2001).

**Table 1:** A noncomprehensive summary of heritability estimates for production, reproduction, and adaptation traits in Angora goats

Breeding objective	Trait	Heritability	Reference
Growth	Weaning weight	0.20	Snyman (2012)
	Weaning weight	0.19	Snyman (2020)
	Weaning weight	0.52	Bolormaa et al. (2010)
	Body weight	0.12-0.58^1^	Snyman (2012)
	Body weight	0.17-0.74^1^	Snyman (2020)
	Body weight	0.55-0.58^2^	Bolormaa et al. (2010)
Reproduction	Lifetime litter weight weaned	0.11	Snyman (2020)
	Lifetime number of kids born	0.14	Snyman (2020)
	Lifetime number of kids weaned	0.10	Snyman (2020)
Nematode resistance	Packed cell volume	0.49-0.59	Bolormaa et al. (2010)
	Faecal egg count	0.02-0.16	Bolormaa et al. (2010)
	Faecal egg count	0.06-0.20^3^	Olayemi et al. (2011)

^1^ Estimates at different ages within one study at 8, 12, and 16 months.

^2^ Estimates at different ages within one study at 6, 12, and 18 months.

^3^ At 3 and 5 months of age respectively, in Cashmere goats.

**Table 2:** A noncomprehensive summary of heritability estimates for production, reproduction, and adaptation traits in Boer goats

Breeding objective	Trait	Heritability	Reference
Growth	Weaning weight	0.23	Menezes et al. (2016)
	Weaning weight	0.29	Garcia-Muniz et al. (2018)
	Weaning weight	0.32	Ball et al. (2001)
	Body weight	0.37-0.45^1^	Ball et al. (2001)
Reproduction	Litter size at birth	0.12	Zhang et al. (2009)
	Litter weight at birth	0.01	Menezes et al. (2016)
	Litter size at weaning	0.10	Zhang et al. (2009)
	Litter weight at weaning	0.10	Menezes et al. (2016)
Nematode resistance	Packed cell volume	0.06	Thomas (2015)
	Faecal egg count	0.13	Thomas (2015)
	Famacha score^©^	0.11	Thomas (2015)

^1^ Estimates at different ages within one study at 8 and 12 months.

From Tables 1 and 2 and the above discussions, genetic progress in the growth traits is feasible when employing more traditional selection methods based on performance measurements, such as best linear unbiased prediction (BLUP) of breeding values. For the difficult to record disease and heat resistance traits, as well as the lowly heritable reproductive traits, direct selection based on genetic markers or the causative genes, or selection based on genomically enhanced breeding values (GEBV) should be considered.

### Genetic markers

The identification of causative mutations and genetic markers associated with traits of economic importance, has been a focus of research in all livestock species for the past two decades. Identification of quantitative trait loci (QTL), was performed first using microsatellite markers and then single nucleotide polymorphisms (SNPs). The use of dense SNP arrays enabled genome-wide association studies (GWAS) and currently whole-genome sequencing techniques are also employed. The literature abound with studies in which potential genes were identified as markers for various reproduction, production, adaptation and disease related traits in livestock (Henkel et al., 2019; Wang et al., 2019; De Lima et al., 2020; Shrivastava et al., 2022; Shaw et al., 2023). These range from studies where the polymorphisms of the proposed gene were validated phenotypically, differentially expressed gene studies, to selection signatures and GWAS studies where associated genes and gene pathways were identified. In Table 3, a summary is given of some of these genes with known polymorphisms that have been validated for the traits related to adaptation under harsh environments, as discussed above, for the Boer goat and Angora goat related breeds found in South Africa. Due to a lack of studies for South African Boer and Angora goats per se, studies on these breeds from other countries were included. No studies on the other South African goat breeds could be found in literature.

**Table 3:** Genes with known polymorphisms that were already validated for adaptive traits in goat breeds found in South Africa

Trait	Breed	Gene symbol	Gene name	Reference
**Reproductive traits**				
Litter size	Boer goat	*GDF9*	Growth differentiation factor 9	Wang et al. (2019)
Litter size	Iranian Angora (Markhoz Goat)	*GDF9*	Growth differentiation factor 9	Ghoreishi et al. (2019)
Litter size	Iranian Angora (Markhoz Goat)	*BMP15*	Bone morphogenetic protein 15	Ghoreishi et al. (2019)
Litter size	Boer goat	*GH*	Growth Hormone	Mishra et al. (2017) De Lima et al. (2020)
Litter size	Boer goat	*GnRHR*	Gonadotropin Releasing Hormone Receptor	Mishra et al. (2017) De Lima et al. (2020)
Litter size	Boer goat	*KITLG*	Receptor tyrosine kinase ligand	Mishra et al. (2017) De Lima et al. (2020)
Litter size	Boer goat	*FSHβ*	Follicle Stimulating Hormone	De Lima et al. (2020)
Litter size	Boer goat	*LHβ*	Luteinising Hormone	De Lima et al. (2020)
Litter size	Iranian Angora (Markhoz Goat)	*THRSP*	Thyroid hormone-responsive	Ghasemi et al. (2022)
Litter size	Boer goat	*INHA*	Inhibin alpha	Wu et al. (2009)
Sperm quality parameters	Boer goat	*FSHβ*	Follicle Stimulating Hormone	Nikbin et al. (2014)
Sperm quality parameters	Boer goat	*LHβ*	Luteinising Hormone	Nikbin et al. (2014)
Sperm quality parameters	Boer goat	*HSP70*	Heat shock protein 70	Nikbin et al. (2018)
**Body weight**				
Weaning weight	Boer goat	*GH*	Growth Hormone	Hua et al. (2009)
**Disease resistance**				
Faecal egg count Packed cell volume	Boer goat	*DRB1 of MHC II*	Dopamine receptor binding 1	Corley & Savage (2015)
Fecal egg count	Angora goat	*IgA*	Immunoglobulin A	Shaw et al. (2023)
Fecal egg count	Various breeds	*IgG*	Immunoglobulin G	Shaw et al. (2023)
Fecal egg count	Various breeds	*IGF-1*	Insulin-like growth factor1	Shrivastava et al. (2022)
Fecal egg count	Various breeds	*IL-2, IL-13, IL-32, IL-33*	Interleukin family genes	Shrivastava et al. (2022)
Fecal egg count	Various breeds	*IFN-γ*	Interferon Gamma	Shrivastava et al. (2022)
Fecal egg count	Various breeds	*IgE*	ImmunoglobulinEG	Shrivastava et al. (2022)
**Coat color**				
Coat color Skin color	Boer goat Angora goat	*KIT*	Receptor tyrosine kinase	Henkel et al. (2019)
Coat color Skin color	Boer goat Angora goat	*ASIP*	Agouti Signaling Protein	Henkel et al. (2019)
Coat color	Markhoz	*MC1R*	Melanocortin receptor 1	Javanmard et al. (2015)

Apart from the genes included in Table 3, other genes associated with some of the reproductive, growth and nematode resistance traits were identified through GWAS and transcriptome studies (Estrada-Reyes et al., 2019; Mahmoudi et al., 2022; Ncube et al., 2022). None of these have, however, been validated against the respective phenotypic performances yet. The genes identified in Table 3 could be used as the basis for marker-assisted selection programs for South African goat breeds after validation of the genes in the local populations.

### Future strategies

It is commonly acknowledged that genetic improvement in all livestock species depends on the accurate measurement of phenotypic data, and where possible, the added benefit of genomic data. In developing countries such as South Africa, with large numbers of noncommercial farmers, the collection of accurate phenotypes in extensive production systems is problematic. This results in the collection of some easy to measure phenotypes, such as weights. However, traits linked to reproduction, survival, and adaptation have largely been ignored, especially in communal and smallholder farming systems. Although the commercial goat breeds have Breeders’ Associations and stud breeders participate in the National Small Stock Improvement Scheme, the uptake of large-scale phenotyping remains low. Even with more cost-effective genotyping becoming more feasible for developing countries, accurate phenotypes are necessary to be able to apply genomics in animal improvement.

The technology that enables the recording of large volumes of phenotypes in a cost-effective, easy manner, is defined as phenomics. It could assist in capturing some of the hard-to-measure traits associated with adaptation, such as fitness and disease resistance traits. Sensors (such as electronic rump-, neck-, or leg-mounted detectors) that can detect behavior patterns could be used for oestrus detection in livestock species. The systematic collection of phenotypes that can address adaptation and welfare will have to be prioritized to make use of the technologies that are currently available for animal improvement. Knowledge on heat tolerance traits in specifically the South African breeds is still lacking. New technologies involving recording of heat tolerance related traits could be employed to fill this gap.

Due to advances in mobile technology, information and communications technology models can possibly assist even small holders to record phenotypes and give them feedback to assist with management decisions (Mrode et al., 2020). However, reliable internet connectivity is necessary for this technology to be applied successfully. Even in the USA, as much as 40% rural farms lack reliable access to broadband (Pérez‑Enciso and Steibel, 2021), indicating that this will pose a serious limitation to the implementation of such technology in South Africa.

As far as genomics is concerned, goal-oriented research should be undertaken with the already identified genes as basis, to validate the association between the alleles/genotypes at these candidate genes and the respective traits in the South African goat populations. Validated markers could then be included in marker assisted selection programs for the respective breeds. This could be implemented where the use of genomic estimated breeding values is not feasible due to difficulty in recording the necessary phenotypic data. In addition, for nematode resistance, more in-depth research into the exact immune-regulatory mechanisms is still required, especially for the South African breeds. This might also lead to the identification of SNPs associated with candidate genes involved in immunoregulatory mechanisms affecting nematode resistance in South African goat breeds.

## Conclusion

The application of new technologies in breeding and selection is under-utilized in goats in general, and especially in South African goats. The knowledge already available on candidate genes affecting traits relating to adaptation to harsh environments should be exploited and expanded through validation of these genes in the South African goat population. Inclusion of the validated markers in selection programs would facilitate genetic progress in herds where phenotypic recording is challenging. The determining factor whether selection will be based on phenotypic indicator traits, phenotypic traits plus genomics, or genetic markers would be the cost of collecting phenotypic data or samples (e.g., nematode resistance) and analyses of the samples, compared to the cost of genotyping. Increased investments by various role players in applying new technology in recording of phenotypic indicator traits (especially for traits related to adaptation), improved pedigree recording and large-scale genotyping would contribute to accelerated genetic progress in the South African goat breeds.

*Conflict of interest statement*: The authors declare that the research was conducted in the absence of any commercial or financial relationships that could be construed as a potential conflict of interest.

## About the Authors

**Carina Visser** is an associate professor at the Department of Animal Sciences, University of Pretoria (UP). She obtained her PhD degree in Animal breeding at the Department of Animal and Wildlife Sciences (UP). She is currently the Vice President of the International Goat Association. Her research focus is primarily on the molecular-based investigation of small stock, and include population genetic studies on local and indigenous breeds and populations. As food security is a burning issue globally, she also focusses on the genetic improvement of especially indigenous livestock species, which can contribute to the mitigation of this problem.

**Margaretha A. Snyman** is a specialist scientist at the Grootfontein Agricultural Development Institute near Middelburg, Eastern Cape in South Africa. She has 36 years of research experience in animal breeding and genetics in Angora goats, wool and dual-purpose sheep breeds. Her field of specialization is quantitative genetics, but for the past 15 years she also became involved in genomic research. Her research focuses on the “Development and evaluation of animal breeding and selection practices” and “Development and evaluation of genotypes.” She is also the manager and project leader of the Grootfontein Biobank for sheep and goat breeds, and its associated projects.
